# Using herbaria to study global environmental change

**DOI:** 10.1111/nph.15401

**Published:** 2018-08-30

**Authors:** Patricia L. M. Lang, Franziska M. Willems, J. F. Scheepens, Hernán A. Burbano, Oliver Bossdorf

**Affiliations:** ^1^ Research Group for Ancient Genomics and Evolution Max Planck Institute for Developmental Biology 72076 Tübingen Germany; ^2^ Plant Evolutionary Ecology Institute of Evolution and Ecology University of Tübingen 72076 Tübingen Germany

**Keywords:** ancient DNA, biological invasions, climate change, habitat change, herbarium, phenology, pollution

## Abstract

During the last centuries, humans have transformed global ecosystems. With their temporal dimension, herbaria provide the otherwise scarce long‐term data crucial for tracking ecological and evolutionary changes over this period of intense global change. The sheer size of herbaria, together with their increasing digitization and the possibility of sequencing DNA from the preserved plant material, makes them invaluable resources for understanding ecological and evolutionary species’ responses to global environmental change. Following the chronology of global change, we highlight how herbaria can inform about long‐term effects on plants of at least four of the main drivers of global change: pollution, habitat change, climate change and invasive species. We summarize how herbarium specimens so far have been used in global change research, discuss future opportunities and challenges posed by the nature of these data, and advocate for an intensified use of these ‘windows into the past’ for global change research and beyond.

## Introduction

**Figure 1:**
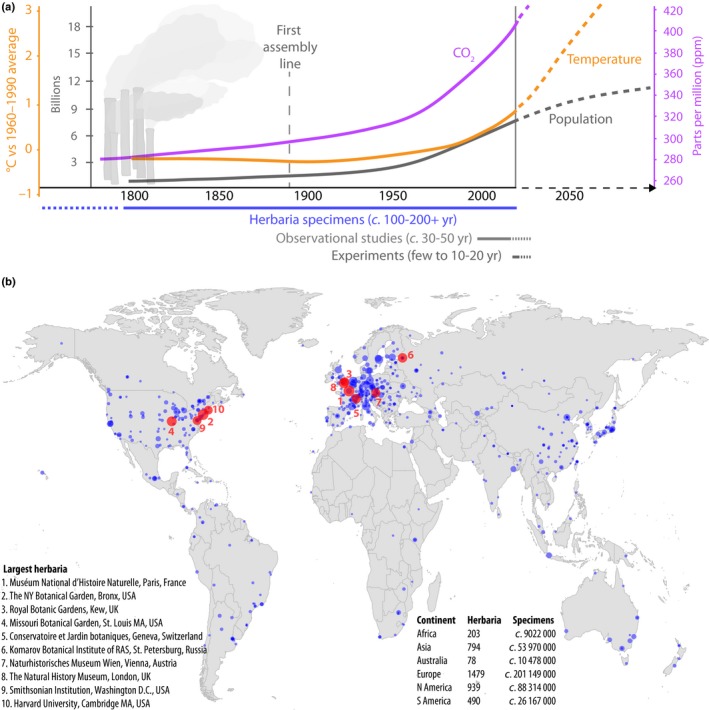
Herbaria as global change witnesses. (a) Timeline of global change, with lines tracking changes in world population, air temperature and atmospheric CO_2_ during the last *c*. 200 years. Dashed line ends indicate future projections. Bars below plot indicate the typical temporal extent of herbarium samples vs observational studies and experiments. (Population growth: United Nations, Department of Economic and Social Affairs, Population Division (2017); World Population Prospects: The 2017 Revision. http://esa.un.org/unpd/wpp/; temperature: representative concentration pathway 8.5, Intergovernmental Panel on Climate Change, www.ipcc.ch; (Marcott *et al*., 2013); CO_2_: (Neftel *et al*., 1994)). (b) Map with global distribution of herbaria (for visual clarity displaying only herbaria of > 100 000 specimens), names of the largest 10 herbaria, and number of herbaria and herbarium specimens curated per continent (reflecting places of storage of specimens, not their origins; Herbarium data from Index Herbariorum, http://sweetgum.nybg.org/science/api/v1/institutions/. Accessed in April 2018).

Global environmental change is one of the major challenges of the 20^th^ and 21^st^ centuries. It has been evident since the age of industrialization in the late 18^th^ century – sometimes also referred to as the advent of the anthropocene – and has continuously gained momentum (Fig. 1a; Steffen *et al*., 2011; Hamilton, 2016). Biologists study global change for its broad ecological impact, and its negative effects on biodiversity. Also, as it represents an unplanned, long‐term and large‐scale experiment, studying global change can promote understanding of fundamental processes such as rapid adaptation. Experimental approaches to study these topics are usually locally focused, and limited to a duration of a few decades (Leuzinger *et al*., 2011). Although observational methods are often more large‐scale and long‐term, they are with few exceptions still restricted to a time frame of 50–80 yr (Fig. 1a; Fitter & Fitter, 2002; Thomas *et al*., 2004). To understand both the extent of global change as a long‐term process, and its full ecological and evolutionary impact, global data that go back to the onset of industrialization are crucial.

**Figure 2:**
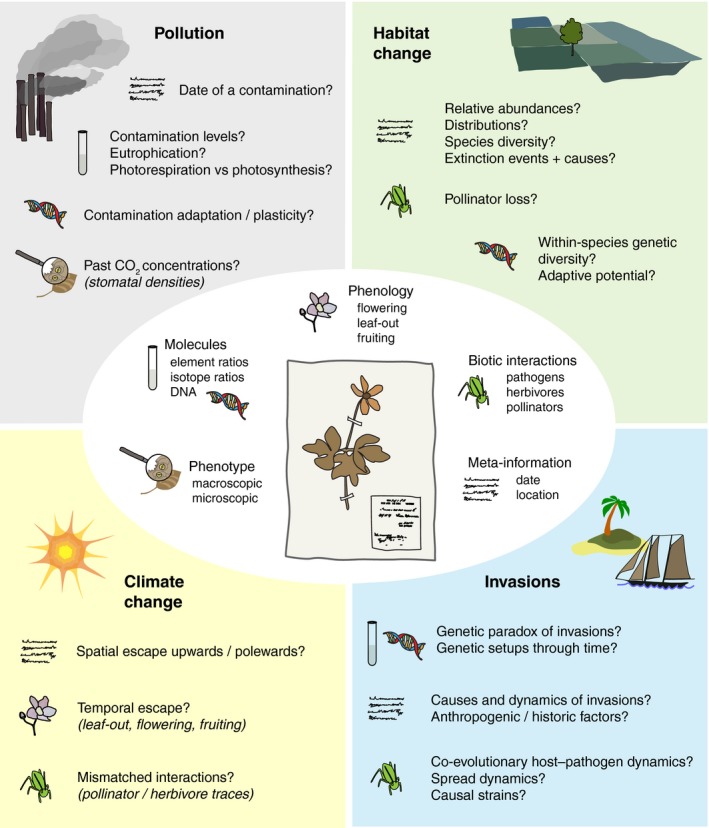
Diversity of herbarium data and their applications. Herbarium sheet in the centre surrounded by types of data that can be obtained from a specimen, with the questions that these data can help to answer around, ordered by respective global change driver. Symbols indicate the type of data used to address each question.

In this context, natural history collections are an underused treasure of temporally and geographically broad samples that we have just begun to dust off (Holmes *et al*., 2016). Especially rich is the botany section of this vault: plants collected, pressed and preserved, in most cases together with meta‐information on species, collection site, date and collector (Fig. 2): In terms of extent, there are > 350 million specimens in almost 3000 herbaria world‐wide (Fig. 1b; Thiers, 2017; http://sweetgum.nybg.org/science/ih/), sampled from the 16^th^ century up to today (Sprague & Nelmes, 1931), and the collections’ potential uses range from classical taxonomy and systematics, to archaeobotany, archaeoecology and climate change research (Funk, 2003). Because plants are sessile, they are particularly exposed to environmental change. The time courses of many of their responses to environmental change are preserved in herbarium specimens, which therefore provide unique spatiotemporal data for studying global change (Primack & Miller‐Rushing, 2009; Lavoie, 2013; Vellend *et al*., 2013; Meineke *et al*., 2018).

Recent studies have emphasized the scientific value of herbaria for a broad range of global change‐related topics (Fig. 2; e.g. Zschau *et al*., 2003; Miller‐Rushing *et al*., 2006; Feeley & Silman, 2011; Willis *et al*., 2017). Dense time‐series of herbarium specimens even permit studying long‐term processes such as recent invasions and their genetic population history (Exposito‐Alonso *et al*., 2018a).

Even though herbaria were used as early as in the 1960s to study global change (e.g. Ruhling & Tyler, 1968, 1969), and are in the process of being made available online via digitization (> 46 700 000 specimens in the Integrated Digitized Biocollections portal alone; as of 18 July 2018 https://www.idigbio.org/portal/ (search terms: type of record – PreservedSpecimen, kingdom – Plantae)), the community has not fully adopted herbaria as valuable ‘time machines’ to the past (Lavoie, 2013; Meineke *et al*., 2018). Especially with the advent of high‐throughput methods and recent technical developments in image analysis, the value of these collections is now more apparent than ever (Munson & Long, 2017).

Simultaneously, next generation sequencing (NGS) techniques now allow for in‐depth genetic analysis of century‐old specimens up to whole genome sequencing of plants and even of their equally preserved pathogens (e.g. Martin *et al*., 2013; Yoshida *et al*., 2013; Durvasula *et al*., 2017; Exposito‐Alonso *et al*., 2018a). This extends the spectrum of available long‐term data far beyond morphology or phenology. For instance, dense sampling of such full genetic information across time – and geography – enables population genetics studies, to follow speciation processes through time, or to quantify changes in genetic diversity in historical contexts. Working with these small samples of degraded DNA – so‐called ancient DNA (aDNA) – retrieved from historic collections is technically challenging and has recently boomed in the animal field (e.g. Shapiro & Hofreiter, 2014; Orlando *et al*., 2015; Marciniak & Perry, 2017), yet in the plant field it is still rarely used (Gutaker & Burbano, 2017).

Here, we present an overview of the different types of herbaria analyses possible in global change research (Fig. 2). Following a timeline from industrialization onwards, we divide herbarium‐related approaches into four main areas related to four main drivers of global change: industrialization causing increased pollution, which coincides with increasing loss of habitat and changes in land use as well as climate change, and finally global trade and transport resulting in an increasing number of invasive species world‐wide. In addition, in excursions dedicated to molecular methods (Box 1), collection biases (Box 2) and the digitization challenge (Box 3), we provide insight into three key methodological issues that herbaria research is currently dealing with, and hopefully inspire with ideas for extended utilization of botanical collections. Our aim is to advocate broader use of herbaria as ‘witnesses’ of global change. We believe that they have the potential to fast‐forward our understanding of the impacts of this unplanned biological experiment, to substantiate our predictions of its long‐term outcomes, and to inform conservation measures.

Box 1 **Molecular analyses and degradation**

The age of herbarium specimens is both their strength and their weakness, as aging is a corrosive process. For most chemicals, the extent, rate and end‐results of this process are not defined in herbarium samples. Still, it is clear that age, but also preservation practices or storage conditions can alter tissue chemical contents. This is evident, for example, when N concentrations measured in stored tissues diverge from the results of previous methods and studies – in this case likely due to post‐collection contamination (Nielsen *et al*., 2017). Hence, in‐depth analyses of correlations between the age and chemical compound quantities in old samples are necessary in order to make claims about historical absolute abundance values (Nielsen *et al*., 2017).

**Figure B1:**
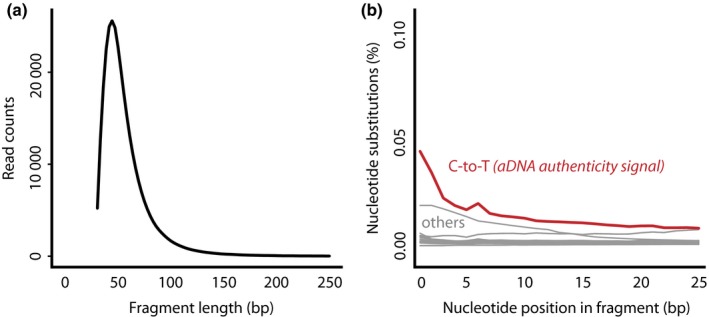
Typical molecular characteristics of herbarium DNA. (a) Fragment size distribution and (b) damage pattern found in ancient DNA (sample data from Weiß *et al*. (2016), publicly available at ENA ID ERR964451).

For DNA from historical samples – aDNA – age‐related degradation dynamics are fairly well‐characterized (Allentoft *et al*., 2012; Weiß *et al*., 2016). Due to chemical modifications, DNA in dead tissue gets increasingly fragmented over time (Fig. B1a), and particularly in fragment ends, aDNA‐characteristic deamination drives nucleotide‐substitutions of cytosine with thymine ((Weiß *et al*., 2016); Fig. B1b). This *per se* does not lessen the potential of aDNA‐studies (Gutaker & Burbano, 2017): specialized protocols even allow extraction of ultra‐short fragments of < 50 bp (Gutaker *et al*., 2017), and the correlation of nucleotide misincorporations with time enables its use as authenticity criterion of ancient DNA (Sawyer *et al*., 2012; Weiß *et al*., 2016). Still, these particular characteristics call for categorical rules for herbarium genetics to minimize contamination risks, verify authenticity and maximize the information gained from precious old plants: samples have to be processed in clean room facilities to avoid contaminations with fresh DNA, and sequenced to a certain depth to yield useful information. Pure PCR analyses on the contrary are inappropriate for aDNA studies, as they do not allow the necessary authenticity verification and, due to the fragmentation of aDNA, are unlikely to yield consistent results.

Such quality requirements are particularly important due to the limitation of available material. Unlike traditional approaches that rely on metadata or morphology of historical samples, molecular analyses require tissue probes and hence destructive sampling of specimens. Therefore, it is the duty of any molecular herbarium scientist to optimize their methods, minimize the amount of sample needed, and employ state‐of‐the‐art analyses to retrieve maximum information from their samples. In the same vein, molecular herbarium scientists and curators should aim to maximize the detail of meta‐information that can be gathered from samples. Knowledge, for example, about temporary field collection in alcohol, or post‐collection specimen treatments with heavy metals (as insecticides or fungicides) is indispensable to assess the suitability of specimens for molecular approaches. Furthermore, both curators and researchers need to assess specimen‐label and specimen‐sample pairs for their correctness, and remain cautious particularly regarding the interpretation of trends in (molecular) data observed only in few or single samples.

Box 2 **Collection biases**

Imbalanced sampling is a well‐acknowledged issue for the use of herbaria, for example, to map species distributions or assess diversity (e.g. Meyer *et al*., 2016; Daru *et al*., 2018). Temporal biases are caused by intense collection periods, and seasonal preferences (Holmes *et al*., 2016). Also, collections often concentrate on easily accessible or much‐frequented sites (geographic bias; e.g. Sofaer & Jarnevich, 2017), and on common or particularly interesting species which – depending on the collectors – can change over time (taxonomic bias; e.g. Feeley, 2012). When working with herbarium data, it is necessary to explicitly test for these biases, for example to avoid a few dominant species generating trends in a dataset (Jácome *et al*., 2007). Depending on the type of question or analysis, biases may need to be corrected for by different means: normalizing collection efforts with different types of reference sets (e.g. Hedenäs *et al*., 2002; Law & Salick, 2005; Case *et al*., 2007), measuring invader distributions in relation to native species (Delisle *et al*., 2003), or verifying trends with additional, nonherbarium datasets (e.g. Lienert *et al*., 2002; Kouwenberg *et al*., 2003; or even those from citizen science, Spellman & Mulder, 2016). In particular when models are based on historical records, comparisons with modern data can support extrapolations or generalizations, but only if biases have been dealt with: models, for example, in the context of invader dynamics and spread, have to take species persistence into account, because historic occurrence does not equal contemporary presence and may cause overestimation of plants’ distribution and abundance (Pergl *et al*., 2012). This is particularly the case for species targeted by eradication measures, such as the human health hazard *Heracleum mantegazzianum*, where herbarium specimens can indicate suitable habitats, but not current occurrence or general invasion dynamics (Pergl *et al*., 2012). Furthermore, there are often no data on early invasion stages, because herbarium records indicate only the presence of a species, whereas its absence is not reliably documented by a lack of records. Conclusions based on modeling and statistical analysis, particularly of early invasion stages, should hence be used as indications rather than be over‐relied upon (Hyndman *et al*., 2015). Finally, the currently rising bias of low collection effort is a well‐known problem for tropical areas (Feeley & Silman, 2011), yet is threatening to become global, via overall declining collections (Prather *et al*., 2004). Although this particularly jeopardizes studies of new or recent invasions (Lavoie *et al*., 2012), it strongly affects all herbarium‐based research.

Box 3 **Digitization challenge**

Large‐scale digitization is crucial to make biodiversity data more accessible, balance the unequal distribution of collections world‐wide (Drew *et al*., 2017; see also locations of all herbaria with > 100 000 specimens world‐wide, Fig. 1b), increase the use of herbaria in general, the number of specimens included per study specifically (Lavoie, 2013), and fuel novel research (see Soltis, 2017; Soltis *et al*., 2018). Various online databases already offer access to vast amounts of data (e.g. https://www.idigbio.org/, www.gbif.org, http://vh.gbif.de/vh/or http://avh.chah.org.au/), but the digitization task is enormous – with over 350 million specimens to process – and expensive. To optimize and speed up the process, various larger and smaller institutions have developed affordable digitization workflows (Haston *et al*., 2012; Nelson *et al*., 2015; Thiers *et al*., 2016; Harris & Marsico, 2017). Depending on data needs, digitization could be done in a prioritized way. In conservation biology, for instance, a fraction of available specimens appears to be enough to reliably detect threatened species and trigger conservation efforts (Rivers *et al*., 2011). How and towards which end such prioritization is carried out, and how large‐scale digitization projects would be funded, is a question that needs to be addressed.

Apart from cost and speed, the transcription of meta‐information, and particularly georeferencing information, is another digitization bottleneck.

Optical character recognition may help sorting entries by collector or country (Drinkwater *et al*., 2014), as might the development of semi‐automated imaging pipelines (Tegelberg *et al*., 2014). Other projects use citizen science approaches to transcribe specimen labels ((Hill *et al*., 2012); https://www.notesfromnature.org/active-expeditions/Herbarium), and computer vision or machine learning (re‐)classify specimens that are unidentified, or whose identification was based on an old taxonomy (Unger *et al*., 2016; Carranza‐Rojas *et al*., 2017; Gehan & Kellogg, 2017). Still, imprecise or wrong georeferencing is common in herbarium data (Yesson *et al*., 2007), an issue that is particularly problematic in conservation, for species distribution assessments, or prediction approaches (Feeley & Silman, 2010). Although care with location data from herbaria is, hence, necessary, digital field notebook apps such as ColectoR may at least help guarantee complete and correct meta‐information for novel collections (Maya‐Lastra, 2016). Finally, in light of concerns about misidentification of up to 50% of tropical specimens world‐wide (Goodwin *et al*., 2015) and the continuously evolving taxonomy, such notebooks, together with the aforementioned computerized identification approaches and even molecular methods, as well as rigorous and continuous manual verification of specimen identities, are crucial to ensure the value of herbaria and herbaria databases.

## Pollution

Technological developments and the mechanization of work in the second half of the 18^th^ century, known as industrialization, changed the landscape world‐wide. Key contributors were improved efficiency of steam engines, the replacement of biofuels with coal and the emergence of a chemical industry. A larger average income, increasing population sizes and accelerated urbanization led to the production of previously unseen quantities of waste and exhausts (Fig. 1a). Herbarium specimens can be used to track historical pollution levels, to serve as a baseline for pre‐pollution conditions, and to connect waste production with species’ reactions – even at the genetic level in the context of local adaptation, or to study long‐term effects of singular events such as the Chernobyl nuclear disaster (Heinrich *et al*. 1994).

### Heavy metals

Metals from the atmosphere, soils and groundwater are deposited on or taken up by plants, and remain present in herbarium specimens, so the latter can be used as indicators of pollution, and due to their meta‐information facilitate the dating of contamination (Lee & Tallis, 1973; Shotbolt *et al*., 2007; Rudin *et al*., 2017). Depending on species, their morphology, physiology and proximity to a pollution source, plants are exposed to and take up more or less pollutants (Lawrey & Hale, 1981; Rudin *et al*., 2017). Studying lead pollution levels, for example, the isotopic lead composition in moss or lichen samples collected at roadsides reflects fluctuations in local motor vehicle traffic, efforts to reduce lead emissions and changes in petrol origin or composition over time (Farmer *et al*., 2002). In addition to lead, herbarium samples also track concentrations of other metals such as cadmium, copper and zinc to follow their temporal and spatial trends in relation to anthropogenic activities (Zschau *et al*., 2003; Shotbolt *et al*., 2007; Rudin *et al*., 2017). Combining pollution records and genetic information from historical and contemporary samples from contaminated sites can even enable studies of plants’ adaptation to pollution at the genetic, heritable level, for example by studying the association between pollution levels and specific alleles, and thus give indications about long‐term adaptation to changing conditions. Such approaches are already well‐established for contemporary data alone (Kawecki & Ebert, 2004; Turner *et al*., 2010; Arnold *et al*., 2016).

### Anthropogenic nitrogen

Similarly, herbaria document human influences on global nitrogen (N) cycling, that started with the rise of the chemical industry and the production of fertilizers, and has peaked since *c*. 1960 (Millennium Ecosystem Assessment, 2005). Moss leaf N‐contents (as well as concentrations of phosphate and sulfur) determined from stable isotope ratios enable inferences about realized N sources and further cycling processes (Peñuelas & Filella, 2001). Such analyses show a retention of additional, anthropogenic N within terrestrial ecosystems (Peñuelas & Filella, 2001). Improved knowledge of these nutrient dynamics within different ecosystems helps us to understand eutrophication. Additional detail on the biotic effects of N fluctuations could be retrieved via shotgun‐sequencing of historical plant roots, given that *bona fide* microbiomes could be recovered, as it has been shown that the bacterial species composition of roots (and soils) is heavily influenced by overabundance of N (Dynarski & Houlton, 2018).

### Increased carbon dioxide

Pollutants such as N or carbon dioxide (CO_2_) can influence overall organismal morphology, making their effects partially measurable without destructive sampling. Increased fossil fuel combustion and the concurrent increase in CO_2_ concentrations since the industrial revolution, for example, correlate with a reduction of stomatal densities on the leaves of herbarium specimens. This trend was already observed in 1987 in a 200‐yr spanning study of woody angiosperm herbaria samples. Further analyses under controlled experimental conditions (Woodward, 1987; Peñuelas & Matamala, 1990) confirm historic samples as proxies to reconstruct past CO_2_ concentrations.

In addition to morphological studies, herbarium specimens enable complementary measurement of global change effects on plant carbon metabolism. Using mass spectrometry to estimate the relative abundances of different carbon isotopes, studies indicate increased water‐use efficiency – the ratio of photosynthesis to water loss – with rising CO_2_ concentrations (Peñuelas & Azcón‐Bieto, 1992; Pedicino *et al*., 2002). With time‐series of genetic variation from herbaria, it is now further possible to determine what part long‐term adaptive changes or phenotypic plasticity play in such physiological or chemical responses.

There is, however, one *caveat* for measurements of any type of chemical compounds in long‐term stored historical samples: Do chemicals suffer degradation processes similar to hydrolytic damages occurring in DNA over time (see Box 1)? If so, to which extent and at what rate do compounds degrade, and what influence do factors like species, specimen mounting or general storage conditions have on such a decay? Systematic studies of chemical degradation through time will permit the assessment of whether absolute or relative values should be used in historical specimens‐based long‐term comparisons.

## Habitat loss and land‐use changes

Apart from pollution, increasing human population densities, urbanization and, in particular, modern agriculture have caused extensive losses, fragmentation or changes of natural habitats. This affects plants’ geographic distribution and densities, for example causing range reductions to more pristine environments (Hallingbäck, 1992). Information about such habitat alterations in response to global change are documented in herbaria. Herbarium sheets normally contain information about the presented species and sometimes other, associated species (referred to in accompanying meta‐information, or co‐sampled with the focal species, e.g. pathogens). Importantly, herbarium sheets also state the time and place of collection. Hence, comparison between past and present localities serves to infer a species’ distribution through time (Hallingbäck, 1992).

### Distribution changes

Many factors have contributed to converting the landscape into a patchwork of agricultural fields, interspersed with cities and roads: industrialization‐associated population growth, urbanization, increasing agricultural acreages due to mechanization of work, or expansion of railroads and other transport systems. Overall, species abundances tend to decrease with habitat and land‐use changes, as is the case, for example, for American ginseng (*Panax quinquefolius*), both as a result of deforestation and of heavy harvesting of wild populations (Case *et al*., 2007). In light of an area's geography, such data also can inform species’ conservation and future trends (Case *et al*., 2007). However, retrospective studies of species’ abundance in a certain location based on historical collections are sensitive both to the quality of available georeferencing data, and to fluctuating collection efforts and other biases (see Box 2). A reference set of specimens picked from the herbarium randomly and independent of species identity can be used to establish a general ‘expected collecting frequency’, which can balance these biases (e.g. Hedenäs *et al*., 2002).

When herbarium records are used to relocate historical populations, current data complement herbarium‐inferred distributions and abundances (Lienert *et al*., 2002; Stehlik *et al*., 2007). Herbaria may in some cases be the only documentation of (likely) extinct species (Chomicki & Renner, 2015). Revisiting surveys can detect such local extinction events, and, in correlation with current land‐use practices or site protection status, be used to study their causes (Lienert *et al*., 2002). They can further document changes in overall plant diversity, which, too, is affected by habitat fragmentation (Stehlik *et al*., 2007). Such approaches are particularly useful to evaluate changes in the local flora and motivate biodiversity monitoring campaigns, and can inform large‐scale diversity surveys, as well as modeling‐based inferences or predictions.

### Indirect effects of habitat fragmentation

Similar to farming‐related landscape changes, urbanization is a prominent driver of biotic interaction changes. One of the most crucial, commercially important types of plant–animal interaction jeopardized, among others, by urbanization and diversity loss, is pollination. Depending on a plant's anatomy, herbaria also house documentation of such interactions, and can illustrate pollinator species decrease or loss. Presence or absence of pollinaria in herbarium specimens of the orchid *Pterygodium catholicum*, for example, reflects the historical pollination rate that depends strictly on a specific bee (*Rediviva peringueyi*) (Pauw & Hawkins, 2011). The bee's decrease following urbanization is consistent with a shift in local orchid communities towards selfing species (Pauw & Hawkins, 2011). Impairment of interactions between plants and their pollinators, caused for instance by such abundance decreases or temporal mismatches, likely also leaves genetic signatures. Given that affected biotic interactions could be identified using historical plant and insect collections, these signatures could be traced through time and inform the potential of other species‐pairs to overcome future mismatches.

Besides the apparent decrease of species diversity, losses of within‐species genetic diversity are a less conspicuous consequence of habitat loss, and are a result of shrinking and increasingly isolated populations (Ellstrand & Elam, 1993; Young *et al*., 1996). Improved high‐throughput sequencing techniques and novel molecular approaches have recently made within‐species genetic diversity – as preserved in herbaria – accessible (see Box 1). This ancient genetic information extends the information on habitat loss and decreasing relative abundances to the genetic level (Cozzolino *et al*., 2007; Martin *et al*., 2014b), with already few specimens giving insights into a population's genetic background. This is crucial knowledge for conservation measures, as genetic diversity, especially in times of increasingly fluctuating environmental conditions, is an indispensable resource for heritable phenotypic variation and rapid adaptation (Huenneke, 1991; Exposito‐Alonso *et al*., 2018b). Reduction of genetic diversity via abrupt decimation of a population, referred to as a bottleneck, can hamper the population's persistence, as selection is less efficient in small populations, where there is more stochasticity and less standing variation to act upon (Ellstrand & Elam, 1993; Young *et al*., 1996; Hartl & Clark, 2007). Comparison of contemporary vs historical genetic diversity can serve to prioritize the conservation of specific populations over others, and to identify genetically diverse source populations for potential reintroductions to balance bottlenecks (Cozzolino *et al*., 2007).

## Climate change

Some factors on the rise since the start of industrialization, and potentially even before that, have less direct, but long‐term effects on ecosystems: the so‐called greenhouse gases such as methane (CH_4_) and CO_2_ (Fig. 1). Their atmospheric increase – for CO_2_ a result of enhanced fossil fuel burning in factories, power plants and for transportation – causes global warming and as a result climate change (Millennium Ecosystem Assessment, 2005). Thus, in addition to the earlier mentioned direct effects of the pollutant CO_2_ on plant morphology and physiology (see the ‘Pollution’ section), progressive CO_2_‐related global warming influences plant life cycles, as is observed for instance already in shifts of plant life cycles, as is observed for instance already in shifts of plant phenology (timing of life cycle events such as flowering and fruiting) to earlier dates. However, herbaria not only directly track these climate‐related plant responses, but also give insights into their ripple‐effects on pollinators, herbivores and even nutrient cycling.

### Range shifts as spatial escape

One possible response of plants to global warming can be distributional shifts when plants escape from unfavorable conditions, which is traceable using herbarium time‐series. Comparison of field with herbarium data verifies predictions that with progressive global warming, species will move both upslope and poleward, following their original climatic niches. For instance, historic time‐series have monitored movements and consecutive diversity shifts in California, Costa Rica and South America as a whole (Feeley, 2012; Feeley *et al*., 2013; Wolf *et al*., 2016), and hence can differentiate successfully moving species from those that may not persist under continuously changing conditions (Feeley *et al*., 2013).

### Phenology timing

Instead of spatial movements, plants also can escape global warming ‘in time’ by shifting phenological events like flowering or fruiting towards more favorable conditions. To track such changes in the past, flowering timing, for example, can be approximated from collection dates of flowering herbarium specimens. Using a combination of contemporary flowering time observations with a herbarium specimen series across > 100 yr and 37 genera, Primack and colleagues (Primack *et al*., 2004) were the first to connect meteorological data with earlier flowering, which was to a great part explained by increasing spring temperatures. This trend has been confirmed by multiple analogous studies (e.g. Davis *et al*., 2015) and also broader approaches that integrated herbarium data with phenology records obtained from field notes and photographs to cover recent years of herbarium record scarcity (Panchen *et al*., 2012).

Spatial scale and statistical power are important factors for these types of studies. Because phenology also depends on latitude, altitude and other environmental factors, broad sampling is necessary to separate climate change effects from other influences. Moreover, as phenology is partly species‐ or plant functional type‐specific, it is useful to study contrasting flowering seasons, native status, pollination syndromes or growth forms (Calinger *et al*., 2013). All of this is facilitated by large‐scale digitization and hence improved accessibility of specimens world‐wide (Lavoie, 2013; Box 3). Such studies, for example, showed that annual plants are generally more responsive to climate change than perennials (Calinger *et al*., 2013; Munson & Long, 2017). Compilation of large cross‐species datasets furthermore allows the search for phylogenetic signals and thus to identify evolutionary processes involved in shaping the observed responses (Rafferty & Nabity, 2017). Apart from interspecies or ‐family variation, plant responses also vary across geographic regions. Combination of world‐wide herbaria allows to capture such responses, enabling to include remote localities across the globe into analyses (Hart *et al*., 2014; Panchen & Gorelick, 2017).

Flowering is not the only phenological event heavily influenced by climate change that can be tracked from herbarium specimens. Depending on a plant's reproductive structures, seed dispersal timing also can be evaluated. At least for the Arctic, dispersal timing, too, seems to advance with increasing temperatures, in correspondence with associated flowering data (Panchen & Gorelick, 2017). Contrariwise, it was also estimated from collection meta‐information (Kauserud *et al*., 2008) that autumnal mushroom fruiting, especially of early fruiting species, is delayed in Norway, possibly reflecting a prolonged growth period due to warm autumn and winter temperatures.

Another parameter that affects entire communities and ecosystem processes is the leaf‐out timing of deciduous trees, as it impacts trophic interactions as well as nutrient and water cycling (Polgar & Primack, 2011). Such data collected from herbarium records track long‐term leaf‐out trends (Zohner & Renner, 2014) and, for example, confirm large‐scale patterns of earlier leaf‐out inferred with satellite data (Everill *et al*., 2014).

### Mismatching biotic interactions

Naturally, these climate change‐related phenomena also affect biotic relationships beyond plants, and hence cannot be seen only as isolated processes. Changes of their timing are likely to affect evolutionarily synchronized relationships, and even their breaking‐up over time is, together with flowering change, partially recorded in herbaria. Combined with entomological museum specimens, herbaria for example document disruption of the plant–pollinator relationship between the bee *Andrena nigroaenea* and the orchid *Ophrys sphegodes* (Robbirt *et al*., 2014). In herbivory relationships, herbarium specimens can actually directly reflect insect reactions to warming. For example, increased traces left by the scale insect *Melanaspis tenebricosa* on maple tree leaves collected in warmer years evidence a higher insect density, perfectly in accordance with observations in the field (Youngsteadt *et al*., 2015). Herbaria can thus help overcome the lack of historical insect abundance records and facilitate evaluation of climate change effects beyond plants alone.

The greatest challenge of most aforementioned approaches investigating species’ responses to pollution, and habitat and climate change, is their inability to distinguish between plastic responses and evolutionary adaptation (Leger, 2013; Munson & Long, 2017), and thus whether observed differences among herbaria specimens reflect genetic changes or just environmentally induced phenotypic changes caused, for instance, by physiological processes (Bradshaw, 1965; Nicotra *et al*., 2010). Quantitative genetics methods using herbarium time‐series could help in disentangling these two alternative hypotheses (Gienapp *et al*., 2008; Tiffin & Ross‐Ibarra, 2014). Once the genetic basis of phenotypic differences is identified, local adaptation can be further tested using traditional approaches such as common garden experiments and reciprocal transplant studies (Savolainen *et al*., 2013).

## Biological invasions

Natural long‐distance dispersal of plants is rare (Nathan & Muller‐Landau, 2000), but as a side effect of global change, plants increasingly move long distances (van Kleunen *et al*., 2015a). This movement massively increased with human migration waves towards the New World in the 16^th^ century, and further accelerated with growing trade and faster transportation – coinciding with the core time range of herbarium collections. Today, jet‐setting plant stowaways establish as ‘neophytes’, ‘aliens’ or ‘invaders’ wherever conditions are favorable enough. With this growing alien species richness, the global species distribution is getting more homogenous (Winter *et al*., 2009). Local plants lose habitats and thus genetic diversity to the invaders, which are therefore considered a threat to biodiversity (Millennium Ecosystem Assessment, 2005).

### Understanding invasion dynamics

Understanding the causes and spatiotemporal dynamics of invasions is indispensable to prevent further damage, preserve natural ecosystems and prioritize management actions (Vilà *et al*., 2011; van Kleunen *et al*., 2015b). Although contemporary surveys depict the current status of invasive species, herbaria track invasions from the first recorded colonizer onwards – which can serve as a proxy, even if it is not the actual first colonizer. In conjunction with contemporary collections and literature surveys, herbaria are crucial to establish inventories of introduced species that monitor their status of naturalization – or invasion – and inform management strategies (Magona *et al*., 2018). With native plants as baseline for collection efforts and abundance, herbaria illustrate geographical and temporal spreads (Crawford & Hoagland, 2009) that may – in search for invasion causes – be connected with historic events. For instance, a map of Chilean alien expansions uncovers two spread peaks, one connected to the spread of agriculture, the other to its increased mechanization (Fuentes *et al*., 2008). Understanding such causalities can feed early preventive measures: retrospectively mapped invasions identify geographic invasion hotspots, and the environmental and anthropogenic factors crucial for their creation. In this way, herbaria can contribute to understanding the general invasibility of particular habitats (Aikio *et al*., 2012; Dawson *et al*., 2017). Furthermore, combined with contemporary data, they can help to identify characteristics of successful invaders, and to quantitatively connect and established naturalization risk with external factors, and rank potential new invaders (Dodd *et al*., 2016).

Herbaria also provide a means of assessing the continued success of invasive species after establishment in a new environment. Previous studies have used them both to predict and to verify predictions of the climatic niche that plants can potentially occupy. For example, the size of the native range of an invasive species has been found to be highly correlated with its abundance in the new range, as documented for many highly invasive Eurasian species around Québec (Lavoie *et al*., 2013). Herbaria also can enable estimation of a weediness index – or how much a plant associates with human‐caused disturbance – which often also overlaps with plant invasiveness (Robin Hart, 1976). Such estimates hold well in comparison with field surveys (Hanan‐A *et al*., 2015). More precise forecasts of a species’ spread can further include its native climate range, again extrapolated from herbarium records, thereby roughly visualizing occupation of a possible climatic niche (Bradley *et al*., 2015). Much as surveying and modeling the dynamics and spread of invaders is crucial to inform containment measures, it is very sensitive to biases and errors in historical collections – one crucial and common error being misidentification and misnaming (Jacobs *et al*., 2017) – and increasingly at risk from decreasing collection efforts (see Box 2).

### Genetic changes of invaders

Irrespective of whether invasive species stay within their native climatic range or move beyond, they face challenges when establishing in new environments. Successful invasive species often adjust to the novel conditions, and it is therefore important to understand such changes in the invasive range.

Adjustment of morphological traits to novel environments is often well‐captured in herbaria, as demonstrated with Australian invasives where 70% of surveyed species showed at least one phenotypic trait changing over time (Buswell *et al*., 2011). With NGS, it is now possible to define whether this trait variation is associated with genomic changes – caused either by drift or potentially adaptive – or more likely the result of phenotypic plasticity. In addition, these methods can potentially solve the ‘genetic paradox of invasion’: the surprising success and spread of colonizers in spite of their reduced genetic diversity (Estoup *et al*., 2016): Do these species adapt based on their (reduced) standing genetic variation, do they borrow pre‐adapted standing variation from native species (adaptive introgression; Keller & Taylor, 2010; Arnold *et al*., 2016), or do they rely on *de novo* mutations and hence novel variation (Exposito‐Alonso *et al*., 2018a)?

Comparison of historic native and invasive populations with contemporary genetic diversity can also point to diversification or hybridization events before species expansion. A recent herbarium genetics study, for example, has shown strong divergences of flowering time genes particularly during the establishment phase of the invader *Sisymbrium austriacum* ssp. *chrysanthum*, possibly enabling a subsequent spread (Vandepitte *et al*., 2014). Such patterns change over the course of invasion. In the Eurasian *Alliaria petiolata* invading North America, invasive success declines along with population age and reduced phytotoxin production in late stages of invasion (Lankau *et al*., 2009). Contrary to that, chemical analyses of herbarium specimens of the phototoxic *Pastinaca sativa*, a European weed also invading North America, displays increased concentrations of phytochemicals over time since invasion, which coincide with the emergence of the herbivore *Depressaria pastinacella* (Zangerl & Berenbaum, 2005). Studies using ancient DNA also have pointed to anthropogenic landscape disturbances causing genetic admixture in *Ambrosia artemisiifolia*'s native populations before its introduction to new habitats, potentially a prerequisite for later invasive success (Martin *et al*., 2014b). In this sense, herbarium material allows us to compare genetic composition through time, and to identify so‐called ‘cryptic’ (i.e. hidden) invasions, where native genotypes are dispelled by phenotypically indistinguishable but more successful and aggressively spreading non‐native relatives (Saltonstall, 2002).

### Hitchhiking invaders: pathogens and herbivores

Moving beyond plant invasions, herbaria even harbor information about hitchhikers traveling with the original plant stowaways, pathogens, purposely or unknowingly sampled together with their hosts (Yoshida *et al*., 2014). Thereby, they track the invasion (success) stories of plant pathogens such as *Phytophthora infestans*, the microbe at the root of potato late blight and the Irish potato famine (Martin *et al*., 2013, 2014a; Yoshida *et al*., 2013). Other preserved pathogens of particular interest for agriculture include rust fungi and downy‐mildew‐causing oomycetes. Herbaria allow identification of causal strains, their genetic characteristics and their tracking to contemporary pathogen diversity. Coupled with host plant analyses, they provide a (genetic) timeline of host–pathogen dynamics to study and illustrate co‐evolutionary principles such as the arms race between hosts and their pathogens. Genetic analysis of such systems can hence provide crucial insight into spread dynamics of pathogens that could have devastating consequences on crop monocultures world‐wide.

Even for invasive herbivores, historic samples may contain a genetic record. The horse chestnut leaf‐mining moth *Cameraria ohridella*, for example, is preserved pressed and dried in leaves of its host plant (Lees *et al*., 2011). Genetics can backtrack the moth's spread from its native Balkan region, and in conjunction with host plant analyses may identify resistant cultivars and biocontrol agents for the invasive pest (Lees *et al*., 2011).

## Conclusions and outlook

Plants preserved in herbaria offer unique perspectives on global change and its consequences, as they are directly affected victims (Fig. 2). Thus, they represent an invaluable temporal, geographical and taxonomic extension of currently available data employed to understand global environmental change, predict its course and inform conservation measures. To fully take advantage of this potential, and to increase and sustain the value of herbaria for the future, three core areas demand particular attention: the maintenance and curation of herbaria including continued collection efforts, the digitization of collections, and herbarium genomics (see also Boxes 1, 2, 3).

Even though many herbaria are already investing in digitization, only a fraction of the *c*. 350 million specimens world‐wide have been digitized so far. Large‐scale digitization would both encourage the use of herbaria for research, and strengthen projects (e.g. Munson & Long, 2017), as studies including digitized material are able to use large sample sizes (Lavoie, 2013). Fast processing of specimens at consistently high data quality is crucial for making digital herbaria truly useful (Yesson *et al*., 2007), as is substantial funding to enable this task and secure databases’ continuity. Yet, even with increased digitization, the actual power of herbaria – for climate change study amongst other types of research – lies in their continuity through time. Despite growing recognition of the value of herbaria, this characteristic is currently threatened by declining collection efforts (i.e. Prather *et al*., 2004; Meyer *et al*., 2016) and a frequent lack of support for herbaria world‐wide. Consequences of reduced data for modeling and other analyses can already be seen in the tropics, where collections are generally sparse (Feeley & Silman, 2011). To maintain herbaria as the treasure they are today, continued and consistent collection world‐wide is essential, especially because they have recently revealed themselves as a browsable repository of genetic variation and diversity. This drastically increases the value of herbaria for climate change research, and for understanding principles of adaptation and evolution in this context. To date, herbaria are still underused in this aspect (Lavoie, 2013), and in particular, high‐quality sequencing data are scarce. With firm guidelines for protocols and quality standards, pointing also to the necessity of DNA preservation‐informed sequencing efforts, this neglect is likely to change in the coming years.

Hence, being aware of the answers herbaria can give if we use the right methods to ask, it is up to us to keep herbaria alive and well, define what we need to know, and start the questioning.

## Author contributions

O.B., H.A.B., F.M.W., P.L.M.L. and J.F.S. developed the ideas for this review; F.M.W. and P.L.M.L. undertook the literature research and P.L.M.L. designed the figures and wrote the paper with input from all authors.
